# When opposites compensate: proton flux crosstalk between envelope antiporters and ATP synthase

**DOI:** 10.1093/plphys/kiaf444

**Published:** 2025-09-26

**Authors:** Ritu Singh

**Affiliations:** Assistant Features Editor, Plant Physiology, American Society of Plant Biologists; Department of Plant Science, University of California, Davis, CA 95616, USA

Photosynthesis underpins life on Earth by converting solar energy into the chemical energy that sustains nearly all food webs. In plants, photons are absorbed by chloroplasts and funneled to the photosystem reaction centers. Electrons excited in PSII travel through the electron transport chain to PSI, producing NADPH, while the cytochrome b6f complex pumps protons into the thylakoid lumen to establish a proton-motive force (pmf). ATP synthase harnesses this pmf to generate ATP, and together ATP and NADPH fuel the Calvin–Benson cycle (Blankenship 2002). However, fluctuating environments frequently challenge this delicate energy balance. When absorbed light energy exceeds the capacity for carbon fixation, the electron transport chain becomes over-reduced, leading to the production of reactive oxygen species. To prevent such damage, plants employ photoprotective strategies, most prominently nonphotochemical quenching (NPQ). The rapid, energy-dependent component of NPQ (qE) is activated by lumen acidification (ΔpH), which drives the xanthophyll cycle and protonates the PSII protein subunit S to safely dissipate excess energy as heat (Ruban 2016).

Maintaining chloroplast energy balance requires precise regulation of proton and ion gradients across both thylakoid and envelope membranes. While ATP synthase provides the main route for proton efflux from the lumen, chloroplast envelope proteins also influence lumen pH and photoprotection. One such envelope protein, DAY-LENGTH-DEPENDENT DELAYED-GREENING1 (DLDG1), is a putative K⁺(Ca²⁺)/H⁺ antiporter whose loss in *Arabidopsis thaliana* causes lumen over-acidification and elevated NPQ (Harada et al. 2019; Suzuki and Masuda 2023). In contrast, the *hope2* mutant, harbouring a single amino acid change in the ATP synthase γ-subunit, shows defective regulation of proton conductivity (gH⁺) and reduced NPQ. These contrasting phenotypes raised the possibility that DLDG1 and ATP synthase might work together to fine-tune proton flux and optimize photoprotection (Takagi et al. 2017; Degen et al. 2023).

In a recent *Plant Physiology* study, Trinh and colleagues (Trinh et al. 2025) investigated the genetic interplay between the envelope transporter DLDG1 and the chloroplast ATP synthase by creating a *dldg1hope2* double mutant. Plants were grown under short-day conditions, and NPQ kinetics were measured across light–dark transitions. A striking partial compensation emerged: the slow, diminished NPQ response of *hope2* was substantially restored in the double mutant, particularly during the first minutes of illumination under low and moderate actinic light. NPQ levels in the double mutant approached those of the *dldg1* single mutant, which is known to exhibit elevated photoprotective quenching.

Since the enhanced NPQ of *dldg1* requires both ΔpH and PSII protein subunit S activity (Harada et al. 2019; Suzuki and Masuda 2023), the authors hypothesized that the rescue observed in the double mutant reflects enhanced ΔpH formation supplied by the *dldg1* background. To probe this mechanism, the authors measured proton conductivity using the electrochromic shift (ECS), a technique that assesses the pmf. The result showed a balancing effect: while *hope2* displayed elevated H+ conductivity (gH⁺) and *dldg1* exhibited reduced gH⁺ and increased pmf, the double mutant had (gH⁺) values closer to wild type. Moreover, the characteristic pmf increase of *dldg1* was absent in the double mutant, supporting the idea of mutual compensation in proton flux. Importantly, western-blot analyses detected no changes in the redox status of the ATP synthase γ-subunit, suggesting that this genetic interaction arises from metabolic adjustments rather than a redox switch.

Since photosystem performance is sensitive to environmental conditions, the authors also compared mutant responses under varying CO₂ conditions and ambient air. Under low CO_2_ conditions, no significant differences in PSII electron transport were observed between wild-type and mutant plants. However, at moderate (400 ppm) and high (1500 ppm) CO_2_, *hope2* mutants showed significantly lower PSII electron transport than *dldg1*. This indicates that *dldg1* and *hope2* mutations exert opposing effects on photosynthetic electron transport under carbon-sufficient conditions. Notably, *dldg1hope2* double mutant did not significantly differ from *dldg1*, indicating that the *hope2*-associated reduction in photosynthetic electron transport activity may be partially complemented by the *dldg1* mutation. Similarly, under ambient air, compared with wild type, *hope2* plants showed reduced NPQ induction and elevated electron pressure at PSII, while these defects were partially alleviated in the double mutant. Photosynthetic performance at both PSII and PSI therefore supports the conclusion that introducing the *dldg1* null mutation into the *hope2* background provides partial functional compensation, mitigating the impaired photoprotection and electron transport of *hope2*.

Would this biochemical compensation benefit plant growth? To address this, the authors compared biomass and photoinhibition under short-day, long-day, constant-light, and fluctuating-light conditions that revealed a more complex picture. Under short days, *dldg1* plants resembled wild type, *hope2* accumulated slightly less biomass in line with reduced CO₂ assimilation, and the double mutant showed only a modest improvement. In long days or constant light, both *dldg1* and *dldg1hope2* developed transient pale-green leaves with reduced maximum PSII efficiency (Fv/Fm) that recovered upon maturation. Most strikingly, under fluctuating light, *hope2* plants were dwarfed and photoinhibited, and the double mutant performed even worse, with a pronounced drop in Fv/Fm. These findings underscore that compensation at the photosystem level does not guarantee enhanced growth under dynamic environments and may even compromise fitness. Interestingly, the authors found that the pale-green phenotype of *dldg1* (and *dldg1hope2*) under constant light was partially alleviated by NaCl supplementation, paralleling the behavior of KEA1/2 antiporter mutants and hinting at shared roles in stromal ion homeostasis.

Collectively, these findings support a model in which DLDG1 influences stromal pH and ionic balance in ways that modulate ATP synthase activity and thereby affect lumen acidification and qE (Fig. 1). In *hope2*, defective metabolic regulation of ATP synthase leads to excessive proton efflux from the lumen; removing DLDG1 shifts stromal conditions to restrict this efflux, restoring ΔpH and enabling NPQ induction. Whether this interplay reflects a direct functional partnership or an indirect effect mediated by other transporters such as KEA1/2 or rapid-activated chloroplast ion channels remains an open and exciting question.

**Figure 1.**
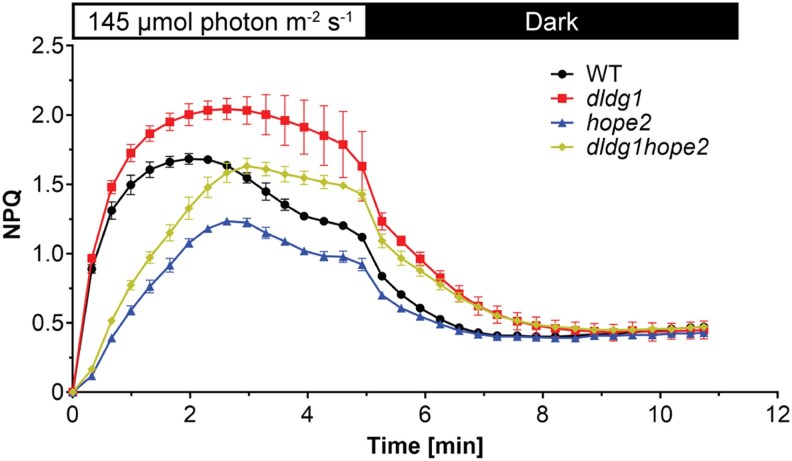
NPQ induction and relaxation in wild-type (WT) and mutant plants (adapted from Figure 1B; Trinh et al. 2025). Plants were dark acclimated for 30 min prior to measurement. A moderate actinic light (AL) intensity of 145 *μ*mol photons m⁻² s⁻¹ was applied for 5 min to induce NPQ, followed by a dark phase to monitor relaxation kinetics. Curves show mean NPQ ± error. Genotypes are WT, single mutants *dldg1* and *hope2*, and the double mutant *dldg1hope2*. The *dldg1* mutant shows the highest NPQ amplitude and slowest relaxation, *hope2* exhibits the lowest amplitude, and the double mutant displays intermediate behavior relative to WT.

This study highlights the remarkable plasticity of chloroplast ion transport networks, where mutations in seemingly distinct components can compensate for one another at the biochemical level. Yet it also underscores the challenge of scaling from molecular adjustments to whole-plant fitness: what stabilizes photoprotection under controlled light may not guarantee improved performance in fluctuating natural environments.

## Data Availability

No data is generated in this study.
